# Comparison of immunity against canine distemper, adenovirus and parvovirus after vaccination with two multivalent canine vaccines

**DOI:** 10.1002/vms3.274

**Published:** 2020-04-27

**Authors:** Rafes D. S. Cunha, Camilo L. da Silva Junior, Camilla A. Costa, Hulliana M. de Aguiar, Danilo G. Junqueira Júnior

**Affiliations:** ^1^ Centro Universitário do Triângulo/UNITRI Uberlândia Brasil

**Keywords:** Brazil, canine viruses, double‐blind method, immunogenicity, vaccination

## Abstract

**Background:**

Viral diseases are a major cause of morbidity and mortality in puppies. There is a belief among veterinary practitioners and even educational institutions that the vaccines made in Brazil against canine distemper virus (CDV), canine parvovirus (CPV) and canine adenovirus (CAV) are ineffective or only partially effective.

**Objectives:**

This study aimed at comparing the immunity of two multivalent vaccines in adult dogs in the city of Uberlândia, Minas Gerais state, Brazil.

**Methods:**

The study was carried out at the Animal Protection Association and a total of 60 adult mongrel dogs were selected and divided into two groups. Group A was immunized with two doses of Elevencell^®^ vaccine and Group B received two doses of imported vaccine from the United States; each group was made up of 14 females and 14 males.

**Results:**

In group A, the Elevencell vaccine generated a protective antibody titre against CDV in 26 out of 28 subjects (92.85%), CPV in 24 out of 28 subjects (85.71%) and CAV in 26 out of 28 subjects (92.85%). In group B, the imported US vaccine generated a protective antibody titre against CDV in 22 out of 28 subjects (78.57), CPV in 21 out of 28 subjects (75%) and CAV in 25 out of 28 subjects (89.28%). There was no statistical difference between titres generated between vaccine types for any of the three diseases tested.

**Conclusion:**

Elevencell vaccine titres were not inferior to the imported US vaccine in conferring protective titres against CDV, CPV and CAH, which confirms the efficacy of this product.

## Graphical abstract

A vaccine made in Brazil is an appropriate immunogen to induce a strong immune response in a highly challenging environment such as the APA shelter

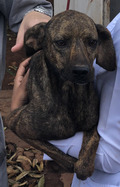

## 1 INTRODUCTION

Viral diseases are a major cause of morbidity and mortality in puppies. In this canine population, there is a higher prevalence of canine distemper, parvovirosis and canine infectious hepatitis (Vila Nova et al., 2018). These three diseases are aetiologically different, but they can be prevented by vaccination with recombinant or live‐attenuated vaccines (Day, Horzinek, Schultz, & Squires, 2016).

Canine distemper virus (CDV) induces several clinical signs, including fever, dyspnoea, diarrhoea and neurological disorders. These signs may vary according to the host immune status and virus strain. Puppies are the most susceptible group to this infection and present the highest fatality rate (Martella, Elia, & Buonavoglia, 2008).

Parvoviruses is caused by canine parvovirus type2 (CPV‐2), characterized by tropism through rapidly dividing cell lines and affecting dogs at different ages. This disease causes a severe enteric infection with bloody diarrhoea, immune suppression and also high fatality rates. The continuous incidence of enteritis is due to the ability of the virus to mutate, which gives rise to new, more resistant and virulent subspecies (Goddard & Leisewitz, 2010).

Canine infectious hepatitis is a systemic viral disease in dogs caused by canine adenovirus type1 (CAV‐1). This virus has tropism for hepatocytes and endothelial cells, which can cause hepatocellular necrosis and systemic bleeding. Unvaccinated puppies are the most susceptible to this infection and present non‐specific clinical signs, which requires differential diagnosis of other diseases such as canine distemper (Decaro, Martella, & Buonavoglia, 2008).

There are several commercially available vaccine brands, with vaccination protocols developed by the manufacturing laboratories or established by scientific research groups such as Comité Latino Americano de Vacunología en Animales de Compañía/Federación Iberoamericana de Asociaciones Veterinarias de Animales de Compañía (COLAVAC/FIAVAC, 2016) and the *World Small Animal Veterinary Association* (WSAVA: Day et al., 2016). Therefore, to choose an appropriate vaccine and the right age for vaccination, it is crucial to seek veterinary advice.

There is a belief among veterinary practitioners or even educational institutions that the vaccines made in Brazil against CDV, CPV and CAV are ineffective or only partially effective. However, there are no published scientific data to support this.

A study carried out in Viçosa, Minas Gerais (Brazil), showed that the facility where vaccination is performed (veterinary clinics or agricultural stores) is not a determining factor for successful immunization, but rather adherence to the schedule recommended (Monti, Viana, Dias, Moraes, & Salcedo, 2007).

The lack of research providing a better understanding of the effectiveness of vaccines made in Brazil may influence the opinion of clinicians and pet owners when choosing the best immunogen. Thus, this study aimed to compare two commercial vaccines, one made in Brazil and another coming from abroad, for efficacy against three diseases, namely: canine distemper, parvovirosis and canine infectious hepatitis.

## 2 MATERIALS AND METHODS

### 2.1 Study design

This study consisted of a randomized double‐blind comparative trial. All procedures were evaluated and approved by the Ethics Committee on the Use of Animals at the Centro Universitário do Triângulo (UNITRI) under the protocol 47/2017‐2.

The data that support the findings of this study are available on request from the corresponding author. The data are not publicly available due to ethical restrictions.

### 2.2 Sample description

This trial was performed at *Associação de Proteção Animal* (Animal Protection Association, APA in short) in Uberlândia, Minas Gerais state, Brazil. APA, an institution founded in 1996,which has a total of 37 housing units divided into three sectors for dogs, as well as a nursery with 10 housing units for cats and dogs plus two catteries, totalling 300 dogs and about 70 cats. These animals were rescued from the streets, where they had been abandoned, abused or injured.

For this study, the criteria for inclusion were animals that had no clinical signs of distemper, parvoviruses and infectious hepatitis, they were dewormed, presented with a medical history inside the shelter (more than a sheltered year) and had negative results in the colorimetric test for the studied antigens. Animals with a change in the physical examination, under the age of 3 or over 10 years, less than a year housed or had positive results in the colorimetric test were excluded.

A total of 60 dogs were selected (sampling error 12%), half of them males and half females. The animals studied were mongrel adult dogs aged between 3 and 10 years that received the same diet plus water (ad libitum) and were housed in the same housing unit.

All animals underwent a thorough physical examination by a veterinarian in order to check for the presence of petechiae, ectoparasites, overt organomegaly and any other abnormalities that could be identified in the examination and interfere with the results.

Randomization was adopted first stratified by sex, by selecting 30 males and 30 females. Then they were separated into blocks of two animals with two sequences of intervention. To guarantee the blinding of the study, the researchers had no contact with vaccines and animals until the moment of the vaccination. The vaccines were stored, prepared and coded by a guest veterinarian who was unaware of the purpose of the experiment. Thus, both animals and researchers were blinded for the protocol used in the vaccination.

At the end, each group was composed of 15 males and 15 females. Group A was given V11 Elevencell Vac (made in Brazil at Labovet^®^) and Group B received immunization with a vaccine imported from the United States (Vangard^®^ Plus, Zoetis Inc.).

### 2.3 Vaccines

One of the vaccines used in this study, brand name V11 Elevencell Vac, contains live‐attenuated virus antigens of distemper, canine parvovirus, infectious hepatitis, adenovirus type2, canine parainfluenza virus, coronavirus‐inactivated antigen and five *Leptospira* serovars (*L. canicola, L. icterohaemorragiae, L. pomona, L. grippothyphosa and L. copenhageni*). The other vaccine used in this study was imported from the United States (Vangard^®^ Plus) and contains four *Leptospira* serovars (*L. canicola, L. pomona, L. gryppothyphosa and L. icterohaemorrhagiae*) as well as live‐attenuated viral antigens of distemper, canine parvovirus, adenovirus type‐2 and canine parainfluenza virus. Both vaccines were stored in the same place and selected because they had a similar formulation with live‐attenuated virus and bacterin.

### 2.4 Sampling and testing procedures

Samples were collected on two occasions: Day0 (also known as D0 or pre‐immunization time point) and Day 42 (D42, post‐immunization period). Each group (A and B) was given two doses of their respective vaccines (Elevencell^®^ or the other vaccine imported from United States, respectively) per animal 21 days apart, following the WSAVA Vaccination Guidelines for non‐vaccinated adult dogs (Day et al., 2016). The schedule was as follows:

- Stage 1: Blood collection and vaccination (Day 0)
- Stage 2: Vaccination (21 days after Stage 1)
- Stage 3: Blood collection (21 days after Stage 2)

Blood samples were collected from the cephalic or saphenous vein and refrigerated for clot retraction, followed by centrifugation and serum separation. Serum was stored at a temperature of −22°C until the tests were performed.

### 2.5 Evaluation of vaccine response

All analyses were performed in a clinical laboratory at UNITRI. The pre‐ and post‐vaccination responses were evaluated using the commercially available kit ImmunoComb^®^ (Biogal Galed Labs) based on solid‐phase ‘dot’‐ELISA technology and designed for detecting serumIgG or IgM levels, validated against gold standard tests: virus neutralization assay(VN) and haemagglutination inhibition assay (HI).

In addition, this test kit is a qualitative and quantitative method that provides a diagnosis within 30 min at room temperature (the best results are obtained at a temperature of 20–25ºC) and consists of: (a)a developing plate with 72 wells containing ELISA test solutions; (b) an ImmunoComb card that is inserted in these wells and immersed in the solutions to determine antibody titre; (c) individually calibrated pipettes of 5.0 and 10.0 μl per sample; (d) CombScale, a one‐colour scale for scoring reaction intensity (i.e. the reading); and (e) tweezers to pierce the wells of the developing plate.

Interpretation of the test results according to the manufacturer uses a colour scale from S0 to S6. There are four levels of interpretation:S0, negative; S1–2,inappropriate immunity; ≥S3,positive; ≥S5,strongly positive. All dogs with a reading equal to or higher than S3 were regarded as immunized or protected. The same titre was used for all three diseases.

The test presented the following values for specificity (Sp) and sensitivity (Se): CAV, Sp 93% and Se 94%; CPV, Sp 100% and Se 88%; CDV, Sp 92% and Se 100% (Biogal Galed Labs Acs Ltd., 2016). The cut‐point S3 indicates a significant response of anti‐CAV antibodies (1:16 titre in VN), anti‐CPV antibodies (1:80 titre in HI) and anti‐CDV antibodies (1:32 titre in VN).

### 2.6 Statistical analysis

The data for the animals were entered individually into Excel spreadsheets (Version 2013; Microsoft Corp.). As the procedure is a scale test with a non‐normal distribution, the median post‐vaccination titre response was obtained, as well as its comparison using the Mann–Whitney non‐parametric test at a significance level of 5%.

Descriptive statistics were used to calculate the frequencies of animals immunized, and proportions were compared using the binomial test for two proportions at a significance level of 5%. All analyses were carried out using BioEstat 5.0 software (Ayres, Ayres Junior, Ayres, & Santos, 2007).

## 3 RESULTS

Of the 60 animals selected and randomly distributed into two groups, only 56 were analysed because three were adopted during the trial and one died as a result of trauma unrelated to enrolment in the study. Thus, each group consisted of 28 dogs.

Before immunization, both groups of animals presented results of ≤2 on the colorimetric scale, which means that all of them were eligible to take part in the vaccination protocol.

When analysing antibody titres against canine distemper, 92.85% (26/28) of the animals of Group A were protected (i.e. with a titre of ≥3)and 78.57% (22/28) of Group B were protected; thus, there was no significant difference between the groups (*p* = .12). Both groups had a median response of 3.5 on the colorimetric scale and again there was no difference between the groups.

With regard to the response against canine parvovirus, Group A was shown to have a protective titre of ≥3in 85.71% (24/28) and Group B in 75% (21/28). There was no statistical difference between the groups (*p* = .31). Both groups had a median response of 4 on the colorimetric scale and again there was no difference between the groups.

For analysis of antibody titres against adenovirus, Group A was shown to have a protective titre of ≥3in 92.85% (26/28) and Group B in 89.28% (25/28); thus, there was no statistical difference (*p* = .63). Group A had a median response of 5.0 for colorimetric titration and Group B showed a median response of 4.0. However, these differences were not statistically significant.

Table 1 shows the frequency of test results for both groups distributed according to the colorimetric scale of the ImmunoComb^®^ kit.

**TABLE 1:** Distribution of ImmunoComb^®^ test kit results according to colorimetric scale by group studied

Canine adenovirus		
Scale	A	B
Canine adenovirus		
1–2	2	3
3–4	9	13
5–6	17	12
Canine parvovirus		
1–2	4	7
3–4	14	12
5–6	10	9
Canine distemper virus		
1–2	2	6
3–4	21	18
5–6	5	4

Note

Scale: 1–2, inappropriate immunity; 3–4, positive; 5–6, strongly positive. All dogs with a reading equal to or higher than 3 were regarded as immunized or protected. All dogs with a reading of 1 or 2 show the presence of some immune memory cells against the virus tested.

## 4 DISCUSSION

Randomized trials are a powerful tool for reducing bias. By distributing the animals randomly into groups, this ensures uniformity between them. Coupled with a double‐blind strategy, this helps to avoid any bias that could favour a particular treatment or control (Oliveira & Parente, 2010).

Although no statistical difference between the two vaccines has been shown, a comparable proportion of animals was protected using vaccine V11 made in Brazil, which reinforces the quality of the product in comparison to the vaccine imported from the United States.

Several different factors can affect vaccine induction of a protective titre and may account for the lack of an appropriate response in some animals: factors such as storage conditions, nutritional status of the animal, maternal antibody titres and vaccine immunogenicity (Day et al., 2016; Monti et al., 2007).

In relation to storage conditions, the vaccines used in this study were stored according to both manufacturers’ guidelines and normative instructions (IMA, 2012) at a temperature between 2°C and 8°C in a cold chamber, which ensures the quality of the products.

Another common cause of vaccination failure involves high levels of maternal antibodies, which can inhibit or neutralize the action of the vaccine (Nandi, Kumar, Mohapatra, & Ravishankar, 2013). However, all animals immunized in this study were adults and therefore there was no correlation between vaccination failure and maternal antibody presence.

Ecto‐ and endoparasites can also influence the effect of the vaccine because these parasites extract nutrients from the host, causing weakness, anaemia, increased stress and secondary bacterial infections (Bowman, Lynn, Eberhard, & Alcarez, 2003). Thirty days before the beginning of vaccination, all animals were given fenbendazole, a broad‐spectrum benzimidazole anthelmintic drug used against endoparasites, and also fipronil for the control of ectoparasites.

Additionally, when selecting the animals for this trial, those that presented with apathy, weight loss, pale mucous membranes, petechiae and ectoparasites were excluded from the study. Every effort was made to control any variables that could interfere with the immune response of each animal individually.

There were limitations to this study that can be addressed in the future. The absence of public and private funding for execution of the project limited the tests that could be carried out, such as complete blood count, imaging tests to evaluate the spleen and liver and also individual quantification of antibodies by spectrophotometry.

## 5 CONCLUSION

Both vaccines are effective in the protection of dogs and the V11 Elevencell Vac made in Brazil has been shown to be an appropriate immunogen to induce a strong immune response in a highly challenging environment such as the APA shelter.

## CONFLICT OF INTEREST

On behalf of all authors, the corresponding author states that there is no conflict of interest.

## AUTHOR CONTRIBUTIONS

**Rafes Dantas da Silva Cunha:** Conceptualization; Investigation; Writing‐original draft. **Camilo Linhares Silva Junior:** Conceptualization; Investigation; Writing‐original draft. **Camilla Amaral Costa:** Conceptualization; Investigation; Writing‐original draft. **Hulliana Machado Aguiar:** Conceptualization; Investigation; Writing‐original draft. **Danilo Guedes Junqueira Junior:** Formal analysis; Project administration; Validation; Writing‐review & editing.

## ETHICS STATEMENT

All authors have been personally and actively involved in the manuscript and are jointly and individually responsible for their content.
